# A Human Osteochondral Tissue Model Mimicking Cytokine-Induced Key Features of Arthritis In Vitro

**DOI:** 10.3390/ijms22010128

**Published:** 2020-12-24

**Authors:** Alexandra Damerau, Moritz Pfeiffenberger, Marie-Christin Weber, Gerd-Rüdiger Burmester, Frank Buttgereit, Timo Gaber, Annemarie Lang

**Affiliations:** 1 Charité—Universitätsmedizin Berlin, Corporate Member of Freie Universität Berlin, Humboldt-Universität zu Berlin, and Berlin Institute of Health, Department of Rheumatology and Clinical Immunology, 10117 Berlin, Germany; alexandra.damerau@charite.de (A.D.); moritz.pfeiffenberger@charite.de (M.P.); marie-christin.weber@charite.de (M.-C.W.); gerd.burmester@charite.de (G.-R.B.); frank.buttgereit@charite.de (F.B.); annemarie.lang@charite.de (A.L.); 2 German Rheumatism Research Centre (DRFZ) Berlin, a Leibniz Institute, 10117 Berlin, Germany

**Keywords:** mesenchymal stem cells, tissue engineering, osteochondral unit, in vitro model, rheumatoid arthritis

## Abstract

Adequate tissue engineered models are required to further understand the (patho)physiological mechanism involved in the destructive processes of cartilage and subchondral bone during rheumatoid arthritis (RA). Therefore, we developed a human in vitro 3D osteochondral tissue model (OTM), mimicking cytokine-induced cellular and matrix-related changes leading to cartilage degradation and bone destruction in order to ultimately provide a preclinical drug screening tool. To this end, the OTM was engineered by co-cultivation of mesenchymal stromal cell (MSC)-derived bone and cartilage components in a 3D environment. It was comprehensively characterized on cell, protein, and mRNA level. Stimulating the OTM with pro-inflammatory cytokines, relevant in RA (tumor necrosis factor α, interleukin-6, macrophage migration inhibitory factor), caused cell- and matrix-related changes, resulting in a significantly induced gene expression of lactate dehydrogenase A, interleukin-8 and tumor necrosis factor α in both, cartilage and bone, while the matrix metalloproteases 1 and 3 were only induced in cartilage. Finally, application of target-specific drugs prevented the induction of inflammation and matrix-degradation. Thus, we here provide evidence that our human in vitro 3D OTM mimics cytokine-induced cell- and matrix-related changes—key features of RA—and may serve as a preclinical tool for the evaluation of both new targets and potential drugs in a more translational setup.

## 1. Introduction

The osteochondral unit is an essential part of the joint and commits the functional association of the articular cartilage, calcified cartilage and the subchondral bone. Its main function is to transfer mechanical strain during weight-bearing and to ensure the mechanical and metabolic homeostasis as well as the overall joint integrity. Articular cartilage is surfacing the subchondral bone, adsorbing mechanical loading and distributing forces within the joint, while the subchondral bone provides mechanical stability, maintains the joint shape and supplies nutrient and oxygen for the deeper layers of the avascular cartilage [1].

Several pathologies have been demonstrated to affect the osteochondral unit e.g., microcracks, microedema, microbleeding, the development of subchondral bone cysts and osteophytes co-localizing with regions of articular cartilage damage [2,3,4]. All these changes are also attributed to the degenerative joint disease osteoarthritis (OA) or chronic autoimmune-mediated joint inflammation such as found in rheumatoid arthritis (RA), which is a systemic autoimmune disease. OA is principally characterized by articular cartilage degeneration often accompanied with subchondral bone erosions due to a higher load impact and the presence of certain mediators and growth factors [5,6]. On the other hand, the progressive, destructive processes in RA are driven by a persistent inflammation of the joint. The complex pathogenesis of RA involves a diverse interplay between various humoral factors, cell types and tissues, though many underlying triggers and mechanism are still unclear. Beside the production of autoantibodies, the release of pro-inflammatory cytokines, such as tumor necrosis factor (TNF)α, interleukin (IL)-1, -6, -17 and macrophage migration inhibitory factor (MIF) and the induction of matrix degrading enzymes such as matrix metalloproteases (MMPs) drives both inflammation and destructive processes within cartilage and subchondral bone leading to an imbalance in metabolic processes [7]. During RA, MMP1, MMP3, MMP8, and MMP13 are predominantly involved in the extracellular matrix remodeling and degradation of cartilage collagens and proteoglycans but may also affect bone (e.g., MMP3, MMP13) [8,9].

As long as the causes of the disease are unknown, current therapies in clinical application aim to reduce the inflammatory mechanisms in the pathogenesis of RA, whereby their unwanted effects with regard to joint and bone homeostasis are often neglected or accepted, as exemplified by the use of glucocorticoids and their pro-osteoporotic effects [10]. According to current recommendations, today’s treatment goal is to achieve remission or at least low disease activity [11]. Despite major progress in the treatment of RA, a strong unmet medical need remains, as not all patients reach the treat-to-target goal, i.e., sustained clinical remission or low disease activity; about 25% still suffer from moderate or even high disease activity [11]. Therefore, preclinical models which reflect the complexity of the functional unit of the joint are essential to improve our understanding of pathophysiological mechanisms, to increase our knowledge on adverse drug effects in clinical use, and to develop and verify new therapeutic approaches.

Until today, animal models represent an integral part of the preclinical drug discovery process. While animals do not develop spontaneously autoimmune conditions such as RA—which constitutes an inherent limitation of these models—arthritis can be induced in these animals by a single agent or by genetic manipulations [12,13,14]. Finally, non-humanized rodent models are not suitable to test treatment strategies which are highly specific for human target proteins [12]. Understanding the homeostasis within the osteochondral unit as well as RA‑related mechanisms is essential for determining treatment strategies. Therefore, different in vitro models have been developed and evaluated during the last years ranging from tissue explants, simplified (co)culture systems and complex tissue engineered three-dimensional (3D) (multi)component systems to chip approaches [14]. Most of the current in vitro cell culture systems in monolayer are used to study the effect of e.g., humoral factors or therapeutics on chondrocytes [15,16], aggregate-cell interactions or cell-cell interplay [14,17], lacking the complexity of (patho)physiologically relevant cell-cell and cell-matrix interactions and nutrient gradients [14,18]. Today, complex 3D in vitro systems include the co-cultivation of e.g., bovine cartilage discs with human synovial fibroblasts mimicking early cartilage destructive processes [19], porcine chondrocytes with an RA-derived cell line [20], RA synovium with bone explants [21] or cartilage explants from either humans or animals [22]. Cartilage and bone differ in matrix characteristics and microenvironmental and mechanical cues. Therefore, osteochondral tissue engineering requires (i) a unique cell and matrix composition, (ii) a certain organization of the artificial tissue with or without scaffold and (iii) specific biological properties. To date, promising in vitro tissue engineering approaches have been developed using (i) scaffold-based bone and scaffold-free cartilage [23], (ii) different scaffolds for both bone and cartilage [24], (iii) a single heterogeneous scaffold [25] or (iv) a single homogenous scaffold for both [26]. A major challenge is the restriction to obtain human primary cells or explants, the limited lifespan of explants [27] and the unstable phenotype of chondrocytes during monolayer expansion [28]. Therefore, mesenchymal stromal cells (MSCs) are often used to engineer cartilage and bone equivalents [14]. Despite major progress especially due to emerging techniques such as 3D bioprinting, so far, there is no appropriate in vitro model which is able to mimic an inflamed joint with respect to the osteochondral unit allowing the preclinical testing of a variety of specific therapeutic approaches.

**Figure 1:**
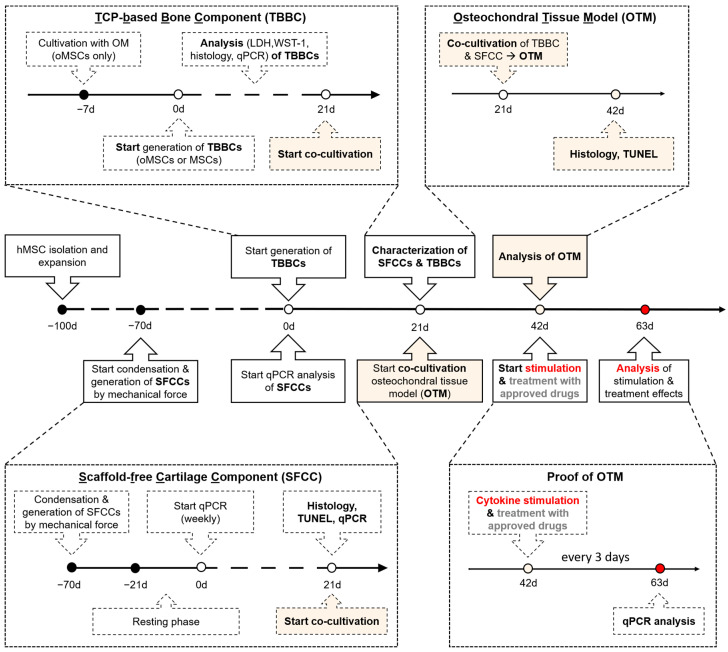
Schematic overview of the timely interlocked processes to generate the human in vitro 3D osteochondral tissue model of arthritis. Based on human bone marrow-derived mesenchymal stromal cells (MSCs), the in vitro 3D TCP-based bone components (TBBCs) and scaffold-free cartilage components (SFCCs) were developed. The osteochondral tissue model (OTM) was engineered by co-cultivation of both tissue components for 21 days. To replicate cytokine-mediated features of rheumatoid arthritis (RA), the osteochondral tissue model was stimulated with typical RA-related cytokines (tumor necrosis factor α, interleukin-6, and macrophage migration inhibitory factor) and finally treated with approved drugs (Proof of OTM). oMSC, one-week osteogenic pre-differentiated MSCs; OM, osteogenic medium; TCP, tricalcium phosphate; LDH, lactate dehydrogenase.

Here, we describe a human in vitro 3D osteochondral tissue model (OTM) as potential part of an artificial joint, comprising a scaffold-free cartilage-like component and a tricalcium phosphate (TCP)-based bone-like component (TBBC). In addition, we aimed at demonstrating that this engineered human OTM can be used as in vitro model to study cytokine-driven cell- and matrix-related changes during osteochondral degradation—key feature of RA. Moreover, we evaluated the feasibility of our OTM by using approved biologics, which prevented these cytokine-related changes. An overview on the experimental setup is given in Figure 1.

## 2. Results

### 2.1. Optimization of the TCP-Based Bone Component Results in a Valid and Sustainable Osteogenic Phenotype Replicating the Subchondral Bone Compartment

**Figure 2:**
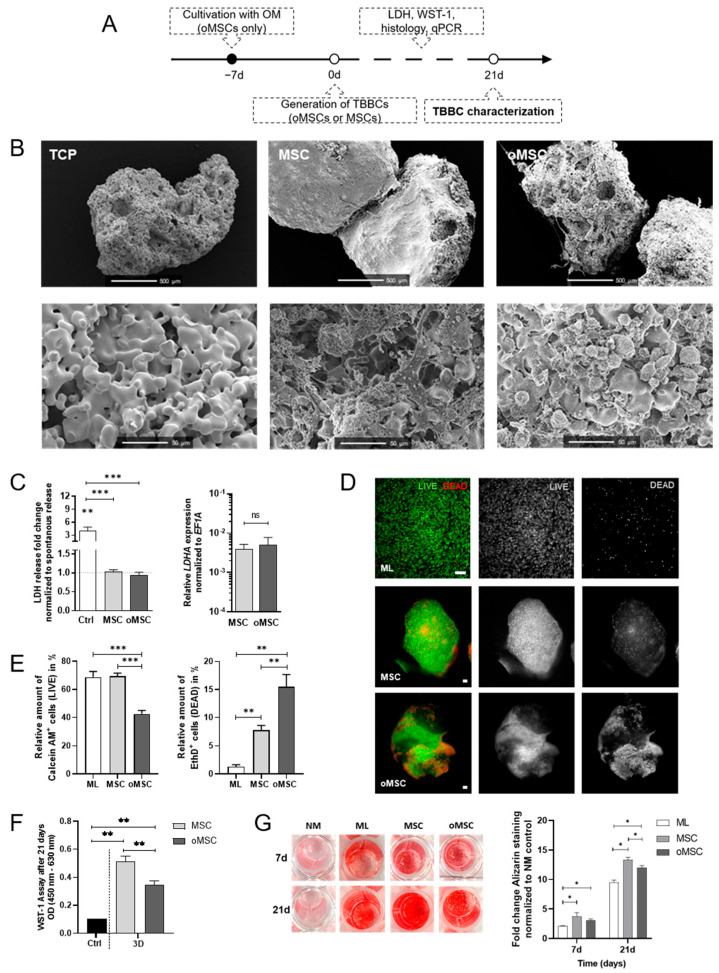
In vitro studies on β-TCP biocompatibility and cell survival comparing the suitability of human mesenchymal stromal cells (MSCs) and osteogenic pre-differentiated MSCs (oMSCs). (**A**) Experimental design of the in vitro TCP-based bone component (TBBC). (**B**) Structural evaluation of β-TCP using scanning electron microscopy. Exemplary images of *n* = 6. Scale bars show 500 µm and 50 µm as indicated in the images. (**C**) LDH-assay was conducted after 24 h to confirm the biocompatibility of β-TCP. Ctrl = 2% of Triton X‑100. Data are shown as mean ± SEM for *n* = 10–12. Gene expression of lactate dehydrogenase A (*LDHA*) was determined by qPCR and normalized to the housekeeper gene *EF1A*. Data are shown as mean ± SEM for *n* = 4. Mann-Whitney U-test was used to determine the statistical significance between groups and Wilcoxon signed-ranked test for the spontaneous LDH release control. (**D**) LIVE/DEAD staining was performed after 21 days and (**E**) quantified using ImageJ. As control MSCs in monolayer (ML) were stained. Green and red colors discriminated between living and dead cells (scale bar = 100 µm). Representative images are shown accordingly for *n* = 4–6. Data are shown as mean ± SEM. (**F**) WST-1 assay was conducted to confirm metabolically active cells after 21 days of 3D cultivation. Ctrl = 2% of Triton X-100. Data are shown as mean ± SEM for *n* = 6. Mann-Whitney U-test was used. (**G**) MSCs and oMSCs were cultivated for 7 and 21 days in normal medium (NM control), in osteogenic medium without β‑TCP (ML) and with β‑TCP populated with MSCs (MSC) or pre-differentiated MSCs (oMSC). Alizarin Red staining was quantified (562 nm). Data are shown as mean ± SEM for *n* = 5. Wilcoxon matched-pairs signed rank test was used to determine the statistical significance. *p*-values are indicated in the graphs with * *p* < 0.05, ** *p* < 0.01 and *** *p* < 0.001 (ns = not significant). TCP, tricalcium phosphate; LDH, lactate dehydrogenase; *EF1A*, eukaryotic translation elongation factor 1 alpha.

**Figure A1:**
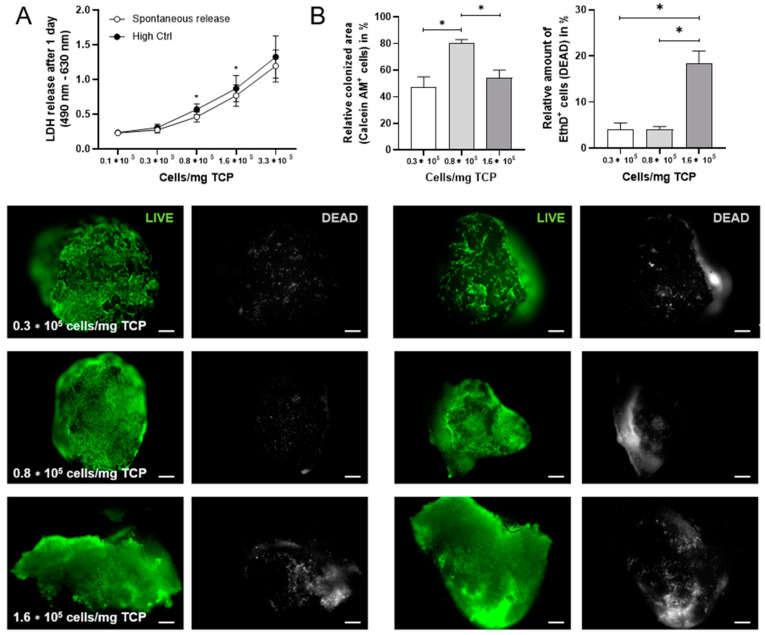
Cell number/mg TCP. (**A**) LDH assay was performed after 1 day and different cell densities (0.1 × 10^5^, 0.3 × 10^5^, 0.8 × 10^5^, 1.6 × 10^5^, 3.3 × 10^5^ cells/mg TCP). High Ctrl = 2% of Triton X-100 to induce LDH release. Data are shown as mean ± SEM (duplicates per donor) for *n* = 4. For statistical analysis the Wilcoxon matched-pairs signed rank test was used; * *p* < 0.05. (**B**) LIVE/DEAD staining was performed after 21 days and different cell densities (0.3 × 10^5^, 0.8 × 10^5^, 1.6 × 10^5^ cells/mg TCP). Images were quantified using ImageJ. Green and grey colors discriminated between living and dead cells (scale bars = 100 µm). Representative images are shown accordingly for *n* = 5. Data are shown as mean ± SEM. For statistical analysis the Wilcoxon matched-pairs signed rank test was used; * *p* < 0.05. TCP, tricalcium phosphate; LDH, lactate dehydrogenase.

Firstly, we determined a cell/TCP ratio of approximately 1 × 10^6^ cells/12 mg (0.8 × 10^5^ cells/mg) as being optimal for the in vitro 3D TCP-based bone component (TBBC) since higher initial cell densities led to increased cell death (Appendix A Figure A1). To test whether osteogenic pre-differentiation of seeded MSCs influences the TBBC formation, we pre-differentiated MSCs using osteogenic differentiation medium for one week (oMSCs) and colonized TCP particles with either MSCs or oMSCs (Figure 2A). Scanning electron microscopy revealed that both MSCs and oMSCs became adherent to the TCP scaffold and invaded the TCP scaffold within 21 days (Figure 2B). Both, MSCs and oMSCs colonized the TCP scaffold within 21 days without any sign of cytotoxicity as demonstrated by the lack of differences in LDH release when compared to the spontaneous release of a TCP-free monolayer (ML), but a significant lower release compared to the positive control (Figure 2C). Analyzing cell viability after 21 days of incubation using LIVE/DEAD staining, we observed a significant lower amount of viable oMSCs (Calcein AM+; green) and an increase in EthD1+ oMSCs (dead, red) when compared to the corresponding MSCs and the respective ML (Figure 2D,E). Analyzing cellular metabolic activity using the WST-1 assay, we detected a significantly reduced metabolic activity in oMSCs compared to MSCs after 21 days (Figure 2F). Interestingly, co-cultivation of MSCs or oMSCs with osteoconductive TCP significantly increased calcium deposition after 7 and 21 days when compared to the monolayer incubated in osteogenic medium but without TCP. Calcification was more pronounced in MSCs than oMSCs (Figure 2G).

Taken together, MSCs and oMSCs colonized the TCP scaffold within 21 days without any sign of cytotoxicity while cell viability, metabolic activity, and calcification were more pronounced in MSCs than oMSCs (Figure 2C–G).

**Figure 3:**
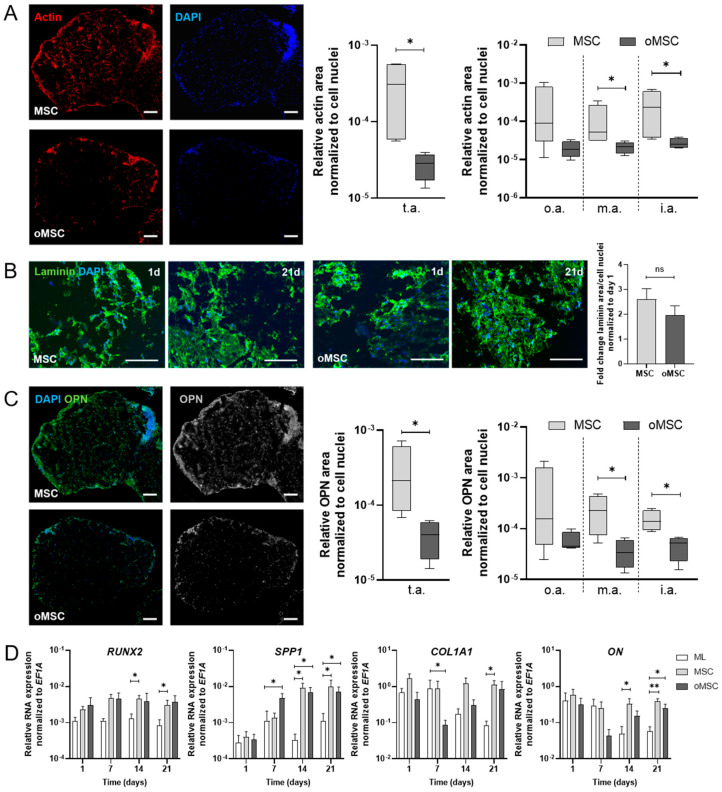
Characterization of the human in vitro tricalcium phosphate-based bone component (TBBC) using both MSCs and oMSCs. (**A**) The amount of actin (in pixels) per cell nuclei (DAPI+) after 21 days within total area (t.a.), outer area (o.a.; outer border determined by a reduction of the diameter by 0.95 for X- and Y-axes), middle area (m.a.; outer area subtracted from total area, diameter reduced by 0.5 for X- and Y-axes) and inner area (i.a.; remaining inner part of the outer area subtracted from total area, diameter reduced by 0.5 for X- and Y-axes) were quantified via ImageJ. Data are shown as Box and Whiskers plot with median ± min/max for *n* = 4. Scale bar indicates 100 µm. (**B**) Laminin and DAPI were stained after day 1 and 21 and quantified via ImageJ. Data are shown as mean ± SEM for *n* = 3. Scale bar indicates 100 µm. (**C**) Osteopontin (OPN) and DAPI were stained after 21 days. The amount of OPN (in pixels) per cell within t.a., o.a., m.a., and i.a. were quantified via ImageJ. Data are shown as Box and Whiskers plot with median ± min/max for *n* = 4. Representative images are shown accordingly (scale bar = 100 µm). (**D**) Total RNA extraction was performed from MSC monolayer (ML) and 3D cultures with MSCs and oMSCs after 1, 7, 14 and 21 days. Gene expression was normalized to the housekeeper gene *EF1A*. Data are shown as mean ± SEM for *n* = 4–6. Mann-Whitney U-test was used to determine the statistical significance; * *p* < 0.05, ** *p* < 0.01 (ns = not significant). MSC, mesenchymal stromal cell; oMSC, one-week osteogenic pre-differentiated MSC; *EF1A*, eukaryotic translation elongation factor 1 alpha.

To investigate the spatial distribution, matrix formation and the osteogenic phenotype of either MSCs or oMSCs on the TCP scaffold, we quantified TBBC sections for the expression of actin, laminin and osteopontin (OPN) normalized to the cell nuclei (DAPI) using immunofluorescence staining (Figure 3A). To assess the spatial distribution, the total area of TBBC section was sub-divided into an outer, middle, and inner area and analyzed for the expression of actin. At day 21, seeded MSCs demonstrate a more pronounced and distributed staining for actin throughout the total area of TBBC section than oMSCs as indicated by significantly more actin staining in the total, middle, and inner area (Figure 3A). To assess the matrix formation, we normalized the relative expression of the extracellular protein laminin as quantified at day 21 to day 1. We found an up to 2-fold increase of matrix production in MSCs and oMSCs over time without observing differences between the two groups (Figure 3B). When focusing on the OPN expression as a measure of osteogenic phenotype, we observed significantly more OPN expression per cell in the TBBCs populated with MSCs than those with oMSCs both in the total area and in the middle and inner area (Figure 3C). Together with the observed actin distribution within the TCP, the OPN results indicate that the invading cells differentiate towards the osteogenic lineage. In this line of observation, the upregulation of runt-related transcription factor 2 (*RUNX2*), secreted phosphoprotein 1 (*SPP1*), osteonectin (*ON*), and collagen type 1 alpha 1 (*COL1A1*), respectively, where superior over time in MSCs as compared to oMSCs (Figure 3D). Based on these findings, we proceeded with the use of MSCs for the generation of TBBCs.

**Figure 4:**
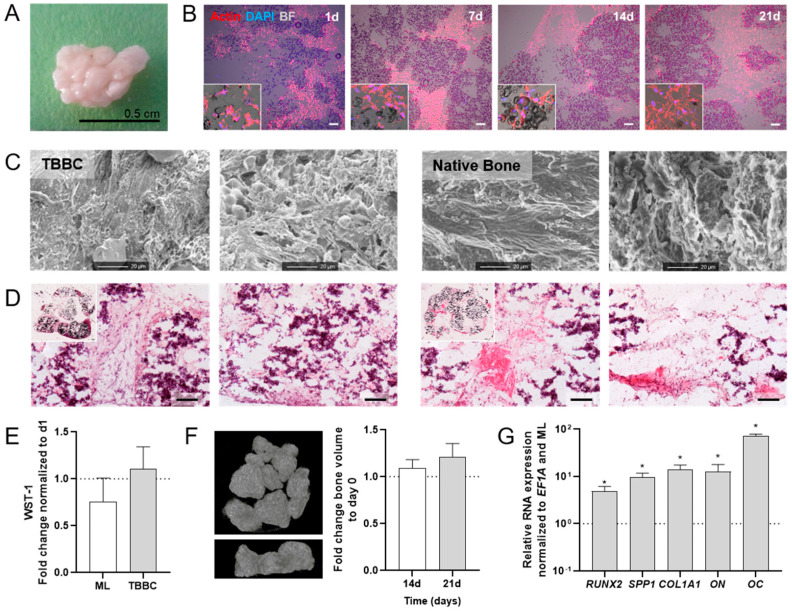
Human in vitro 3D TCP-based bone component (TBBC) based on MSCs. (**A**) Macroscopic overview of the in vitro 3D TBBC. (**B**) Exemplary images to highlight cell localization and extracellular matrix formation as shown by immunofluorescence staining for actin (red) and DAPI (blue) after day 1, 7, 14 and 21. Bright field (BF) shows the β-TCP scaffold. Exemplary images of *n* = 4 (scale bar = 100 µm). (**C**) Structural examination of the TBBC in comparison to native bone using scanning electron microscopy. Exemplary images of *n* = 8 TBBC and *n* = 2 human native bone. (**D**) Histological evaluation of the morphology via H&E staining. Exemplary images for *n* = 8. Scale bars indicate 100 µm. (**E**) WST-1 assay was conducted to confirm metabolically active cells after 21 days of cultivation compared to day 1 and monolayer control (ML). Data are shown as mean ± SEM (duplicates per donor in two independent experiments) for *n* = 6. Mann-Whitney U-test was used to determine the statistical significance. (**F**) In vitro 3D µCT reconstruction and quantitative results. Data are shown as mean ± SEM for *n* = 3. (**G**) Total RNA extraction was performed from ML and TBBCs after 21 days of osteogenic differentiation. The relative gene expression was normalized to the housekeeper gene *EF1A* and osteogenic differentiated ML. Data are shown as mean ± SEM (duplicates per gene) for *n* = 6. Wilcoxon Signed Rank Test was used to determine the statistical significance; *p*‑values are indicated in the graphs with * *p* < 0.05. MSC, mesenchymal stromal cell; TCP, tricalcium phosphate; *EF1A*, eukaryotic translation elongation factor 1 alpha.

The final TBBCs were produced in sizes with a diameter up to 0.5 cm and cultivated for 21 days (Figure 4A). Exemplary images of actin and DAPI immunofluorescence staining over time (day 1, 7, 14, 21) further supported prior endpoint analyses demonstrating the invasion of cells into the TCP scaffold (Figure 4B). Furthermore, TBBCs cultivated for 21 days displayed a cell and matrix formation comparable to native bone as analyzed by scanning electron microscopy (Figure 4C). H&E staining clearly indicated a matrix formation, the interconnection between cells and the TCP particles, and the beginning of osteoid formation at day 21 (Figure 4D). Additionally, seeded MSCs maintained their metabolic activity over 21 days when compared to day 1 and the respective monolayer control (ML; Figure 4E). Analyzing bone formation using µCT, we observed a numerical increase in bone volume at day 21 compared to day 0 (TCP only) and day 14 (Figure 4F). Finally, gene expression of bone specific markers revealed a significant upregulation of *RUNX2*, *SPP1*, *ON*, *COL1A1* and osteocalcin (*OC*) as compared to the osteogenic differentiated ML (Figure 4G). In summary, using MSCs to produce TBBCs is valid to achieve a sustainable osteogenic phenotype and to recapitulate the subchondral bone compartment in our OTM approach.

### 2.2. Characterization of the Scaffold-Free Cartilage Component Demonstrates a Valid Cartilage-Like Phenotype Including Zonal Organization

**Figure 5:**
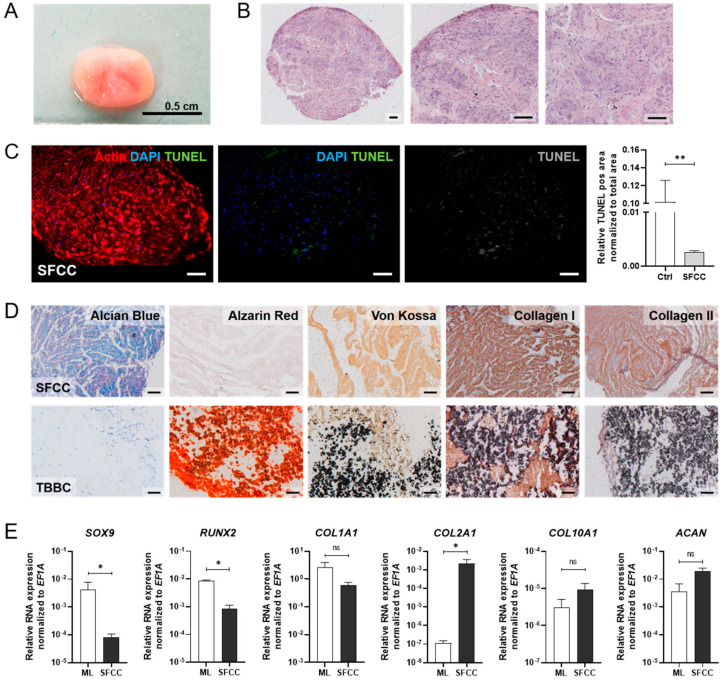
In vitro studies on the 3D scaffold-free cartilage constructs (SFCC) based on human MSCs. (**A**) Macroscopic overview of the in vitro 3D SFCCs. (**B**) Histological evaluation of the morphology via H&E staining. Exemplary images for *n* = 6. Scale bars indicate 200 µm. (**C**) Detecting apoptotic cells (green) using TUNEL staining after 21 days without mechanical force. Exemplary image for *n* = 4. Scale bars indicate 200 µm. Pos. ctrl = incubation with DNase I for 10 min. (**D**) Histological (Alcian Blue, Alizarin Red, von Kossa) and immunohistochemistry staining (collagen type 1 and collagen type 2) of the SFCC in comparison with the tricalcium phosphate-based bone component (TBBC) control. Exemplary images for *n* = 4. Scale bars indicate 200 µm. (**E**) Total RNA extraction was performed from 3D cultures after 21 days. Gene expression was normalized to the housekeeper gene *EF1A*. Data are shown as mean ± SEM (duplicates per gene) for *n* = 3–6. Mann-Whitney U-test was used to determine the statistical significance; *p*-values are indicated in the graphs with * *p* < 0.05, ** *p* < 0.01 (ns = not significant). MSC, mesenchymal stromal cell; *EF1A*, eukaryotic translation elongation factor 1 alpha.

**Figure A2:**
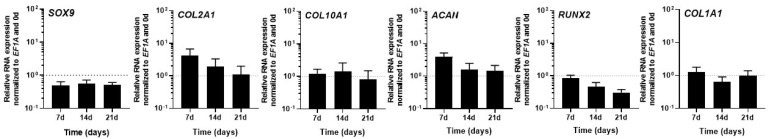
Gene expression analysis of scaffold-free cartilage components for *SOX9*, *COL2A1*, *COL10A1 ACAN*, *RUNX2* and *COL1A1* were performed after 0, 7, 14 and 21 days. The relative gene expression was normalized to the housekeeper gene *EF1A* and day 0. Data are shown as mean ± SEM (duplicates per gene) for *n* = 3. Mann-Whitney U-test was used to determine the statistical significance. *SOX9*, SRY-box transcription factor 9; *COL2A1*, collagen type II alpha 1; *COL10A1*, collagen type X alpha 1; *ACAN*, aggrecan; *RUNX2*, runt-related transcription factor 2; *COL1A1;* collagen type I alpha 1; *EF1A*, eukaryotic translation elongation factor 1 alpha.

The cartilage-like component was produced as described previously. In short, the procedure is based on mesenchymal condensation and the cyclic application of biomechanical force which finally leads to the self-organized 3D scaffold-free cartilage component (SFCC) [18]. Thus, we were asking the question whether we can maintain a cartilage-like phenotype of the SFCCs for 21 days, which is the time point that coincides with the start of co-cultivation with TBBCs (Figure 1). SFCCs were produced in a diameter sized up to 0.5 cm (Figure 5A). H&E staining of histological sections demonstrated an almost homogenous cell matrix distribution with a higher cell density and more flattened cells in the outer surface area similar to the superficial zone of native cartilage (Figure 5B). Additionally, the inner area was characterized by spherical and randomly oriented cells reflecting characteristics of the middle zone of native cartilage. Using TUNEL staining at day 21, we observed only a low number of apoptotic cells within the SFCC (Figure 5C). Histochemistry and immunohistochemistry revealed the presence of glycosaminoglycans (Alcian blue staining) and collagen type 2 (Figure 5D). We also found collagen type 1 expressed in the tissue, although its extent of expression was clearly lower as compared to the TBBC control. However, mineralization was not present as shown by Alizarin and von Kossa staining (Figure 5D). Comparing the gene expression of SFCCs to undifferentiated MSCs in ML at day 21, we observed an up-regulation of cartilage specific markers such as collagen type 2 alpha 1 (*COL2A1*), aggrecan (*ACAN*) and collagen type 10 alpha 1 (*COL10A1*), while the expression of the bone specific markers *COL1A1* and *RUNX2* was downregulated as compared to the monolayer control (Figure 5E). In addition, the cartilage specific transcription factor *SOX9*, which is an early marker of chondrogenesis during cartilage development, was also downregulated (Figure 5E). However, the temporal course of the analyzed marker gene expression revealed no significant changes over 21 days as assessed weekly (Appendix A Figure A2). Based on our findings, we continued with the in vitro generated SFCCs displaying a stable chondrogenic phenotype and characteristics of native cartilage.

### 2.3. Co-Cultivation of Scaffold-Free Cartilage Components and In Vitro 3D Tricalcium Phosphate-Based Bone Components Lead to Formation of a Subchondral Bone-Like Zone

**Figure 6:**
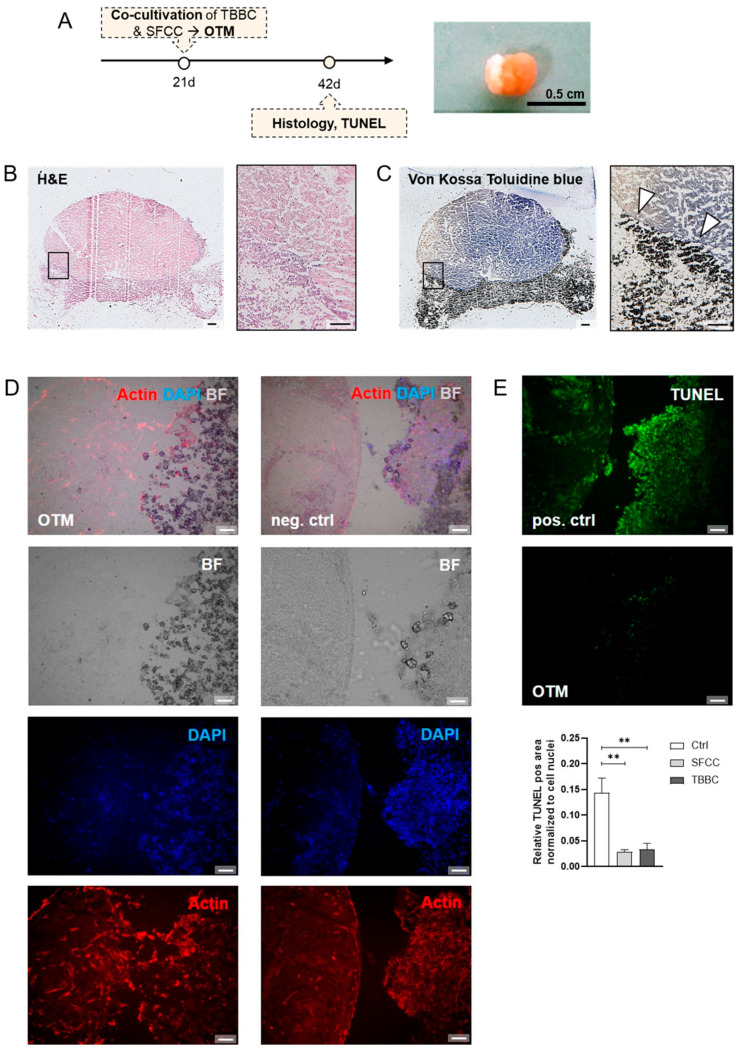
Human in vitro 3D osteochondral tissue model (OTM). Both components were developed independently and subsequently co-cultured in a cell culture insert for 21 days. (**A**) Experimental design of the in vitro 3D OTM. (**B**) Histological evaluation via H&E and (**C**) Toluidine blue combined with von Kossa staining. Exemplary image for *n* = 4. Scale bars indicate 500 µm. White asterisks highlight initial mineralization. (**D**) Actin and DAPI staining were performed to visualize the transitional bridging area between both components. Neg. ctrl = co-cultivation for 1 day, simulating a not unified OTM. Exemplary image for *n* = 4. Scale bars indicate 200 µm. (**E**) The number of apoptotic cells (green; TUNEL staining) normalized to cell nuclei (DAPI+) in both TBBC and SFCC after 21 days of co-cultivation was quantified via ImageJ. Exemplary images for *n* = 5. Scale bars indicate 200 µm. Pos. ctrl = incubation with DNase I for 10 min to induce apoptosis. Mann-Whitney U‑test was used to determine the statistical significance compared to the control, *p*‑values are indicated in the graphs with ** *p* < 0.01. TBBC, tricalcium phosphate-based bone component; SFCC, scaffold-free cartilage component.

Since we aimed to develop a complex human in vitro 3D OTM to mimic the part of a joint which is affected during the late stages of RA, we cultivated the SFCC on top of the TBBC. Therefore, TBBCs and SFCCs were produced as outlined before (Figure 1 and Figure 6A) and cultivated for 21 days before analysis (day 42). H&E staining of OTMs showed that SFCC and TBBC were sticking together without any gap formation (Figure 6B). Although both parts could still be discriminated morphologically by H&E staining, we additionally phenotypically discriminated the SFCC from the TBBC part using Toluidine blue combined with von Kossa staining visualizing the chondrogenic phenotype by Toluidine blue dye attaching to the negative charges of the proteoglycans while calcified tissue phenotype was confirmed by von Kossa (Figure 6C). Moreover, we observed a rearrangement of the cytoskeleton after 21 days of co-cultivation in the bridging area between both components reflecting a subchondral bone-like zone of the osteochondral tissue as visualized by actin and DAPI (cytoskeleton, nucleus) staining (Figure 6D). To confirm cellular viability after 21 days of co‑cultivation, we conducted a TUNEL staining (green: apoptotic cells) showing only a few apoptotic cells compared to the positive control with induced apoptosis by DNase I treatment (Figure 6E). Taken together, co-cultivation of SFCC and TBBC led to the formation of a connecting OTM with cellular rearrangements in the bridging area.

### 2.4. The Osteochondral Tissue Model Shows Cell- and Matrix-Related Changes after Cytokine Stimulation Which Were Prevented by Application of Anti-Rheumatic Drugs

**Figure 7:**
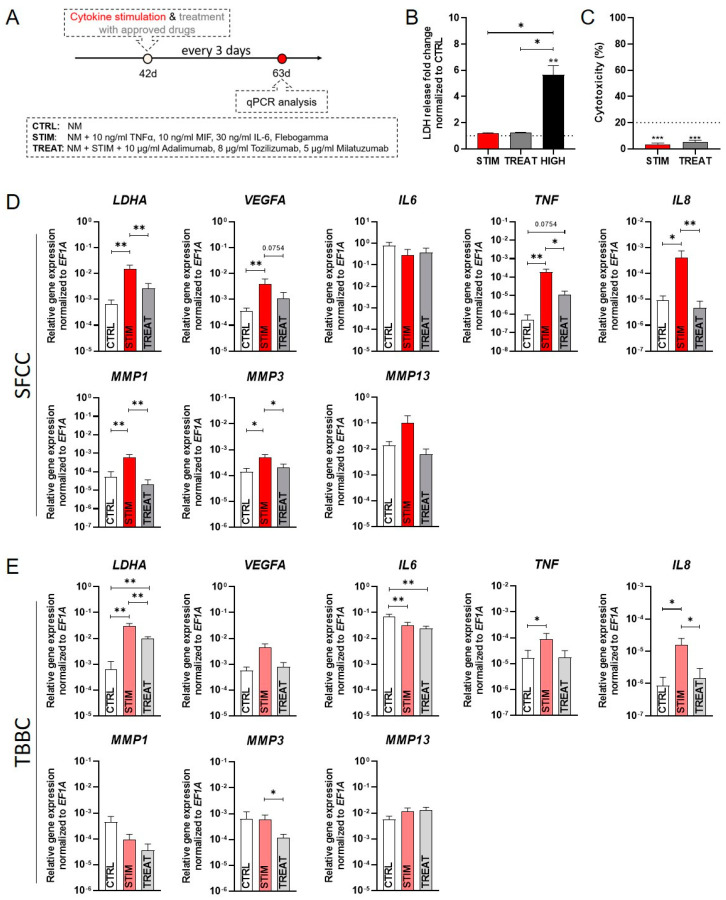
Experimental results of our osteochondral tissue model incubated for 21 days under non-inflammatory conditions (CTRL), repetitive cytokine stimulation with a cocktail of TNFα, IL-6 and MIF (STIM) and under treatment conditions (TREAT). (**A**) Schematic overview of the experimental design. (**B**) LDH-assay was performed after 1 day to cover any cytotoxic effects using supernatant from the OTM treated with cytokines (STIM—10 ng/mL TNFα, 30 ng/mL IL-6, 10 ng/mL MIF) and in combination with clinically available drugs (TREAT—10 µg/mL adalimumab, 8 µg/mL tocilizumab, 5 µg/mL milatuzumab). CTRL = untreated control; HIGH = 2% of Triton X-100 to induce LDH release. Data are shown as mean ± SEM for *n* = 5. Wilcoxon matched-pairs signed rank test was used to determine the statistical significance between groups and One sample t test for the spontaneous LDH release control (CTRL). (**C**) Percentage of cytotoxicity was determined by the following equation [%] = (exp. value − CTRL)/(HIGH − CTRL) × 100 for *n* = 5. Data are shown as mean ± SEM. One sample t test was used with a cytotoxicity cut off of 20% [29]. (**D**) Gene expression studied via qPCR for *LDHA*, *VEGFA*, *IL6*, *TNF*, *IL8*, *MMP1*, *MMP3* and *MMP13* for scaffold-free cartilage components (SFCCs) and (**E**) tricalcium phosphate-based bone components (TBBCs). Data were normalized to the housekeeper gene *EF1A* and are shown as mean ± SEM (duplicates per gene) for *n* = 5. Mann-Whitney U-test was used to determine the statistical significance, *p*‑values are indicated in the graphs with * *p* < 0.05, ** *p* < 0.01, *** *p* < 0.001. LDH, lactate dehydrogenase; TNF, tumor necrosis factor; IL, interleukin; MIF, macrophage migration inhibitory factor; *LDHA*, lactate dehydrogenase A; *VEGFA*, vascular endothelial growth factor A; MMP, matrix metalloproteases; *EF1A*, eukaryotic translation elongation factor 1 alpha.

To mimic the chronic inflammatory environment of RA, our osteochondral tissue model was treated for a prolonged period of 21 days repetitively (every 3 days) using a cocktail of three major RA-related cytokines, namely TNFα (10 ng/mL), IL-6 (30 ng/mL) and MIF (10 ng/mL) at pathophysiological, non-cytotoxic concentrations (Figure 7A–C). Cytokine concentrations were used as reported from synovial fluid of patients with RA (STIM) or left untreated (CTRL). The cytokine cocktail was applied with or without drugs to quantify both the effects of the cytokines and the preventive potential of the drugs under investigation. To this end, we added a combination of clinically available drugs in their therapeutic dosage (10 µg/mL adalimumab, 8 µg/mL tocilizumab, 5 µg/mL milatuzumab; TREAT). After sustained repetitive treatment for 21 days mimicking chronic inflammation, we analyzed the transcriptional response of our model and identified a significant cytokine-mediated upregulation of the metabolic marker lactate dehydrogenase A (*LDHA*) in the STIM group compared to CTRL which was significantly reduced in both SFCC and TBBC when treated with the combination of chosen drugs (TREAT; Figure 7D,E). Additionally, a cytokine-induced upregulation of the angiogenic marker vascular endothelial growth factor A (*VEGFA*) was observed, which was prevented in the TREAT group. The expression of *IL6* was numerically reduced after both cytokine stimulation (STIM) and therapeutic treatment (TREAT) as compared to CTRL in SFCCs (Figure 7D) but was significantly reduced in TBBCs (Figure 7E). Conversely, *IL8* and *TNF* were significantly upregulated after stimulation, while this effect was prevented by the drugs. In the SFCC model, *MMP1* and *MMP3* were significantly upregulated in the cytokine-treated group compared to the CTRL group (Figure 7D). This effect was reversed in the drug-treated group compared to the STIM group. There were no significant differences in gene expression for *MMP13* within the experimental groups. However, in the bone-like model, *MMP1* expression was numerically diminished in both the STIM and TREAT group compared to the CTRL group (Figure 7E). There was no significant difference in gene expression of *MMP3* within CTRL and STIM, while we observed a significant downregulation in the TREAT group. Moreover, there were no significant differences in gene expression for *MMP13* within the experimental groups. Of note, cartilage and bone component differentially responded to cytokine-mediated stimulation with respect to the significantly upregulated expression of *MMP1* and *MMP3*.

In summary, stimulation with TNFα, IL‑6 and MIF lead to cytokine-mediated cartilage degradation, a key feature of arthritis. These findings are in accordance with in vivo data from animal studies and with observations made in human pathophysiology. Cultivation in the presence of immunomodulatory drugs did sufficiently prevent these cytokine-induced changes.

## 3. Discussion

Until today, no appropriate human in vitro model exists so far, which can appropriately mimic the (patho)physiological relevant environment of a healthy or an inflamed joint specifically focusing on the later stage of disease involving cartilage matrix degradation and subchondral bone erosion with the need for a crosstalk between bone and cartilage within the osteochondral unit. Simulating such features in a preclinical drug screening tool would be transformative for the translational process necessary for optimizing rheumatological care. Here, we have developed a human MSC-derived OTM and described its capacity to create a RA-like phenotype, which replicated in part the immunomodulatory effect of well-known anti-rheumatic drugs such as adalimumab [30,31] and tocilizumab [32,33], but also the antineoplastic agent milatuzumab [34].

As in our current study, MSCs are widely used as suitable cell source to engineer in vitro tissue owing to their availability, isolation simplicity, high proliferation rate and differentiation capacity towards individual resident cell types of bone and cartilage [35,36]. Since there has been evidence that high-density scaffold-free chondrogenic cultures and ceramic bone scaffolds might lead to the formation of well-integrated OTMs [37], we firstly engineered bone components by using TCP ceramic particles, which mimic the mineral bony part. Comparable in vitro and in vivo approaches to promote osteogenesis and bone regeneration have been published during the last decade [38,39]. With respect to the biocompatibility and prominent osteoconductive activity of TCP [40], our results (Figure 2 and Figure 3) are in line with a study conducted by Herten et al. where both MSCs and osteoblasts were cultivated on TCP particles [41]. Additionally, we confirmed the osteogenic phenotype based on upregulated expressions of early osteoblast-specific genes, including *RUNX2*, *SPP1* and *COL1A1* and enhanced expression of mature osteoblast markers (*SPP1*, *ON*), indicating the mineralization capacity of MSCs within the tissue models (Figure 4) [42,43]. Secondly, in order to generate cartilage-like tissue components, emerging tissue engineering approaches use mesenchymal condensation based on cell-sheet formation, self-assembly or self-organization to engineer scaffold-free constructs [44,45,46,47]. Although MSCs are well-known to differentiate into various lineages, their capacity to form mature chondrocytes and full articular cartilage is limited. However, chondrogenic differentiated MSCs still provide sufficient similarities to articular cartilage which renders them eligible to serve as therapeutic option for e.g., cartilage defects [48] or in vitro models [18,49]. Furthermore, Li et al. showed that adding mechanical load additionally promotes chondrogenesis of MSCs by up-regulating TGF-β. An approach which is identical to the procedure we applied here. As a result, chondroblastic cells form a functional network, embedded into their self-synthesized matrix comprising both superficial and middle zone and abundant expression of collagen type 2 but also collagen type 1 (Figure 5). The latter is a prerequisite for chondrogenic MSC differentiation, because MSCs firstly reside within the pre-cartilaginous matrix rich in collagen type 1 inducing cell-cell contact, which finally results in an increased expression of *SOX9* and differentiation [50,51,52].

The crosstalk between bone and cartilage within the osteochondral unit is supposed to play an important role in the etiopathogenesis of cartilage matrix degradation and subchondral bone erosion and needs, therefore, to be considered in emerging therapeutic strategies and preclinical testing tools. To mimic the osteochondral unit, common in vitro approaches use a bi-layered scaffold where chondrocytes or MSCs are embedded in polymers and the bone layer is based on e.g., ceramics [53]. Both layers are most often fixed by adhesives such as fibrin resulting in a barrier for cell-cell contacts. Conversely, Lin et al. encapsulated iPSCs-derived MSCs in a gelatin scaffold and cultivated these scaffolds in a dual-flow bioreactor [54]. In contrast to our approach, they directly generated a stable bridging zone between the components but neglected the different cellular demands on the given ECM (stiffness, matrix composition) and microenvironment (oxygen supply) [53]. As shown in our study, co-cultivation for 21 days in a static culture system led to the formation of a bridging zone, suggesting a functional interplay between the two tissue components (subchondral bone-like zone; Figure 6).

Studies have shown that synovial fibroblasts and different immune cells (e.g., neutrophils, macrophages, T‑cells) are a major source of pro-inflammatory cytokines in RA. For example, TNFα, IL-6 and MIF activate resident chondrocytes to produce TNFα, IL-8, IL-6 and matrix metalloproteases (MMP1, MMP3, MMP13) promoting cartilage degeneration and subsequent bone erosion. In line with our results, Pretzel et al. analyzed cartilage degradation of bovine cartilage discs co-cultivated with human synovial fibroblasts and supplemented IL-1β and TNFα. They were able to demonstrate an upregulation of tissue-degrading enzymes (MMP1, MMP3) and pro-inflammatory cytokines (IL‑6, IL-8). In addition, several studies report an increased production of matrix metalloproteinases (MMP3, MMP13) in chondrocytes and cartilage explants after IL-6 treatment, which is in accordance with our results (Figure 7) [55,56,57]. Moreover, TNFα and IL-6 have been recently shown to have overlapping and synergistic effects, even though some of these effects are regulated by separate mechanisms [58]. Interestingly, Honorati et al. analyzed chondrocytes from non-inflammatory pathology in comparison with RA chondrocytes showing that inflammation seems to play an important role in inducing the chondrocyte-related VEGF secretion [59]. Here, we demonstrate that exposure to TNFα, IL-6 and MIF [60,61,62] does induce arthritic transformation followed by activation of the angiogenic marker *VEGFA* in the SFCC (Figure 7) [63]. In contrast, we did not observe a cytokine-related upregulation of *IL6* which has been already described after TNFα exposure. It was shown that the self-activation of pY‑STAT3 by newly synthesized IL-6 leads to a transient auto-inhibition of further IL-6 transcription [64]. Thus, the supplementation of IL-6 may be responsible for the absence of induced IL-6 expression in our study.

Finally, we provide first evidence that effects of immunomodulatory drug candidates can be demonstrated in our MSC-derived OTM on mRNA level (Figure 7). We used adalimumab [30,31], tocilizumab [32,33] and milatuzumab [34] for proof of concept, as these are clinically used monoclonal antibodies directed against TNFα, IL-6 and MIF. Recent work has indicated that TNFα may mediate its angiogenic effect in RA via IL-8 and VEGF [65]. In fact, treatment strategies aimed at decreasing TNFα resulted in decreased angiogenic IL-8 production in vitro and decreased serum levels of angiogenic VEGF in RA patients [66,67]. We reported that treatment in a pharmacologically relevant dose does prevent pro-inflammatory effects, attenuating the TNF and IL-6 signaling pathway. Preclinical testing of therapeutics towards RA is a major challenge, since current therapeutic approaches target specific molecules or pathways by e.g., antibodies or small molecules which are unique in humans. Nevertheless, recent studies have indicated that there are differences in functionality and binding capacity between the human and murine system leading to the potential failure of promising antibodies during clinical trials as exemplified by anti-IL-17 antibodies (e.g., secukinumab) [68] as an biological disease-modifying antirheumatic drugs or the phosphodiesterase 4 inhibitor apremilast [69] which were both tested successfully in mice but failed to provide clinical efficacy in patients suffering from RA [70,71,72].

Nevertheless, the model has still some limitations that need to be addressed in the future. So far, the TBBC does not include bone remodeling processes and our current version of the OTM omits circulating cells such as leukocytes and endothelial cells. However, there is the opportunity to expand our model by including e.g., osteoblasts/osteoclasts, selected RA-related leukocyte populations or human umbilical vein endothelial cells. Finally, an approach that offers possibilities to investigate the cellular behavior and intercellular interactions in a perfused 3D context is given by the organ-on-a-chip technology [73].

## 4. Materials and Methods

### 4.1. MSC Isolation, Cultivation and Characterization

**Figure A3:**
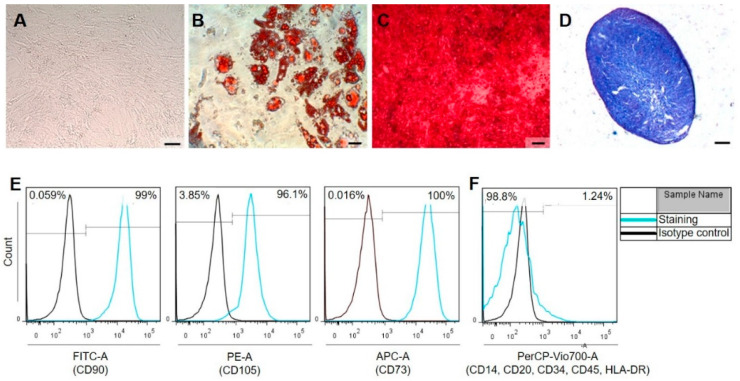
Bone marrow-derived mesenchymal stromal cells are characterized (**A**) by plastic adherence, by their differentiation capacity towards (**B**) adipogenesis using Oil Red O staining, (**C**) osteogenesis using Alizarin Red staining (scale bars = 100 µm), (**D**) chondrogenesis using Alcian Blue staining (scale bar = 200 µm), (**E**) the expression of surface marker CD90, CD105, CD73 and (**F**) lack of CD14, CD20, CD34, CD45 and HLA-DR.

Primary human mesenchymal stromal cells (MSCs) were isolated from bone marrow obtained from patients undergoing total hip replacement (provided by the Center of Musculoskeletal Surgery, Charité-Universitätsmedizin Berlin, donor list in Table 1). Study design and protocols were approved by the Charité-Universitätsmedizin Ethics Committee and performed according to the Helsinki Declaration (ethical approval EA1/012/13, 31 January 2013). Briefly, bone marrow was transferred into a T‑175 flasks (Greiner Bio-one International GmbH, Kremsmünster, Austria) with Dulbecco’s Modified eagle minimal Essential Medium (DMEM) GlutaMAX™ (Gibco, Waltham, MA, USA) supplemented with 10% fetal calf serum (FCS, Biowest, Nuaillé, France), 100 U/mL penicillin (Gibco, Waltham, MA, USA), 100 µg/mL streptomycin (Gibco, Waltham, MA, USA) and 20% StemMACS^TM^ MSC Expansion Media Kit XF (Miltenyi Biotech, Bergisch Gladbach, Germany). After incubation for two days (37 °C, 5% CO_2_), medium was changed, remaining tissue parts were removed, and the adherent cells were washed with phosphate-buffered saline (PBS; pH 7.4). Reaching 90% confluency, cells were passaged. MSCs were characterized after three passages using differentiation assays (adipogenic, osteogenic, chondrogenic) and flow cytometry (MSC Phenotyping Kit; Miltenyi Biotech, Bergisch Gladbach, Germany; CD90+, CD105+, CD73+, CD14−, CD20−, CD34−, CD45−, and HLA-DR−, Appendix A Figure A3). Only MSCs successfully passing the characterization were further used for experiments until passage 3–8.

For differentiation, MSCs were seeded at a density of 1 × 10^4^ cells per well in a 96-well plate (Greiner Bio-one International GmbH, Kremsmünster, Austria) and cultivated in appropriate differentiation medium for three weeks (37 °C, 5% CO_2_). For adipogenic differentiation, MSCs were incubated in StemMACS^TM^ AdipoDiff medium (Miltenyi Biotech, Bergisch Gladbach, Germany). After 3 weeks, cells were fixed in 4% paraformaldehyde (PFA; Electron Microscopy Sciences, Hatfield, PA, USA) for 15 min. After washing with 60% isopropanol, cells were stained with 60% Oil Red O solution dissolved in ddH_2_O (Sigma-Aldrich Chemie GmbH, Munich, Germany: freshly prepared and passed through a 0.45 µm filter; stock solution: 0.3% Oil Red O dissolved in 100% isopropanol) for 15 min, washed with 60% isopropanol. Finally, ddH_2_O was added and intracellular lipid droplets were analyzed microscopically.

For osteogenic differentiation, MSCs were cultivated in StemMACS^TM^ OsteoDiff medium (Miltenyi Biotech, Bergisch Gladbach, Germany) for three weeks. Alizarin Red staining was performed according to the following protocol: cells were washed with PBS, fixed with 4% PFA for 15 min, washed with PBS and stained using a 0.5% Alizarin Red S staining solution (pH 4; Sigma-Aldrich Chemie GmbH, Munich, Germany) dissolved in ddH_2_O for 15 min. After the final washing step with ddH_2_O, calcium deposition was analyzed microscopically.

For chondrogenic differentiation MSCs were transferred in a 3D state (pellet cultivation) via centrifugation for 10 min at 400× *g* and cultivated in StemMACS^TM^ ChondroDiff medium (Miltenyi Biotech, Bergisch Gladbach, Germany). Slices were prepared and Alcian Blue staining was performed to analyze the presence of acidic mucopolysaccharides microscopically.

### 4.2. Generation of the Osteogenic Component—TCP-Based Bone Component (TBBC)

The 3D cultivation was performed in cell culture inserts (Sarstedt AG, Nümbrecht, Germany) with a 0.3 cm^2^ growth area and a polyethylene terephthalate (PET) membrane (8 µm pore size). Approximately 12 mg of β‑tricalcium phosphate (TCP) granules (Cerasorb^®^M, Curasan AG, Kleinostheim, Germany) were preincubated with 1 mL of DMEM GlutaMAX™ supplemented with 10% FCS, 100 U/mL penicillin, 100 µg/mL streptomycin, in the following referred to as normal medium (NM) for one day. To optimize the 3D TCP-based bone component (TBBC), 1 × 10^6^ MSCs or pre-differentiated MSCs (pre-incubated in osteogenic differentiation medium for one week, oMSCs) were seeded onto TCP granules. Cell suspension and TCP particles were gently mixed and cultured in osteogenic medium (OM, NM supplemented with 0.5 mM ascorbic acid, 10^−8^ M dexamethasone, 10 mM L‑glycerophosphate). Medium was changed twice a week.

### 4.3. Generation of the Scaffold-Free Cartilage Component (SFCC)

The 3D scaffold-free cartilage components (SFCCs) were generated based on a patented method (No. 10 2004 001 225, German Patent and Trade Mark Office, 2004) purchased from the Research Center of Medical Technology and Biotechnology (fzmb GmbH), Bad Langensalza, Germany. Briefly, approximately 6 × 10^6^ MSCs were detached and transferred into a 3D state via centrifugation for 10 min at 400× *g*. After 5 to 7 days, biomechanical forces were applied for 3 to 4 weeks to the self-assembled 3D component. SFCCs were then cultivated for up to 21 days without biomechanical forces (resting phase) in NM supplemented with 9.39 mg/L ascorbic acid (Sigma-Aldrich Chemie GmbH, Munich, Germany), in the following referred to as chondrogenic medium (CM).

### 4.4. Generation of the Osteochondral Tissue Model and Experimental Setup

An overview on the experimental setup is given Figure 1. Co-cultivation of both SFCC and TBBC was performed in cell culture inserts by placing the SFCC on top of the TBBC. Chondrogenic medium was added and both components were cultured for 21 days at 37 °C, 5% CO_2_ to develop the osteochondral tissue model by allowing cell-cell interaction. To mimic chronic cytokine-mediated joint inflammation, stimulation was performed using CM (CTRL) supplemented with 10 ng/mL recombinant human macrophage migration inhibitory factor (MIF) [60], 30 ng/mL recombinant human IL-6 [62], 10 ng/mL recombinant human tumor necrosis factor alpha (TNFα; all ImmunoTools GmbH, Friesoythe; Germany) [61] using concentrations as reported from synovial fluid of patients with RA and 23 µg/mL Flebogamma (Grifols, Barcelona, Spain) in the following referred as STIM. To evaluate the impact of specific therapeutic approaches, CM was supplemented with cytokines and well-known clinically available drugs in their therapeutic dosage: 10 µg/mL adalimumab (Amgen, Thousand Oaks, CA, USA) [30,31], 8 µg/mL tocilizumab (Roche, Basel, Switzerland) [32,33] and 5 µg/mL milatuzumab (Immunomedics, Morris Plains, NJ, USA) [34], in the following referred to as TREAT. Medium was changed every 3 days including the respective supplements resulting in a repetitive chronic cytokine stimulation in case of STIM and a repeated counter-treatment in case of TREAT.

### 4.5. Viability and Cytotoxicity Assay

Cell Proliferation Reagent WST-1 Kit (Sigma-Aldrich Chemie GmbH, Munich, Germany) was used according to the manufacturer’s instructions. Samples were mixed with WST-1 solution and incubated for 30 min at 37 °C, 5% CO_2_. Supernatants were measured photometrically using a standard 96-well plate reader (Synergy^TM^ HT Reader, BioTek Instruments, Bad Friedrichshall, Germany) at a wavelength of 450 nm (reference wavelength 630 nm). To induce cell death, a control group of cells was incubated with 2% Triton X-100 (Sigma-Aldrich Chemie GmbH, Munich, Germany) for one day (Ctrl). The assay was performed in two independent experiments with duplicates.

Cytotoxicity Detection LDH Kit (Sigma-Aldrich Chemie GmbH, Munich, Germany) was used to detect cytotoxic effects of TCP particles, cell/TCP ratio, cytokine and treatment concentrations. According to the manufacturer’s instructions the OD-values were measured at a wavelength of 490 nm (reference wavelength 630 nm). Additionally, to induce LDH release via cell death, cells were incubated with 2% Triton X-100 for one day, in the following referred as high control (Ctrl). The LDH assay was performed in duplicates.

LIVE/DEAD^®^ Viability/Cytotoxicity Kit (Invitrogen AG, Carlsbad, CA, USA) was used to determine cell viability and to visualize 3D cell colonization. Samples were first washed with PBS, transferred to a slide and subsequently incubated with LIVE/DEAD^®^ staining solution (established concentration: 2 µM Calcein AM, 4 µM EthD-1) for 35 min at RT in the dark. Evaluation was performed with the fluorescence microscope BZ-9000 (Keyence, Itasca, IL, USA).

### 4.6. Alizarin Red Assay

For calcium quantification, Alizarin Red assay was performed. MSCs were seeded at a density of 1 × 10^4^ cells per well in a 96-well plate and cultivated in NM (NM control), in OM without TCP (ML) and in OM with TCP co-cultivating MSCs (MSC) or pre-differentiated MSCs (oMSC). To exclude TCP-related staining, the OD value of TCP cultivated in OM without cells was subtracted from the TCP OD value. Medium was removed and cells were fixed in 4% PFA (10 min) and stained with Alizarin Red S staining solution (10 min), washed with ddH_2_O, staining was dissolved with 10% cetylpyridiniumchlorid and OD‑values were measured with a plate reader at a wavelength of 562 nm (reference wavelength 630 nm). Data were normalized to the NM OD‑values. The assay was performed in duplicates.

### 4.7. Scanning Electron Microscopy

First, samples were washed twice with PBS and then fixed with 2.5% glutaraldehyde (Sigma-Aldrich Chemie GmbH, Munich, Germany) solved in PBS (10 min, room temperature—RT). After washing with PBS, samples were dewatered using ascending ethanol concentrations 30%, 50%, 70%, 80%, 90%, 95% and twice 100% for 5 min each. Afterwards samples were incubated with hexamethyldisilazane (1 × 5 min, 2 × 10 min; Sigma-Aldrich Chemie GmbH, Munich, Germany). Finally, samples were air dried overnight and coated with gold using a Fine Coater JFC‑1200 (Jeol GmbH, Freising, Germany). The imaging with the scanning electron microscope JCM-6000 Plus Neo Scope^TM^ (Jeol GmbH, Freising, Germany) was performed under high vacuum.

### 4.8. Histochemistry

Slices were prepared using the Kawamoto method to allow the embedding of samples without decalcification [74]. Samples were first fixed in 4% PFA for 3 h followed by an ascending sucrose solution treatment (10%, 20% and 30%) for one day each at 4 °C. Afterwards, the samples were embedded with SCEM embedding medium (Sectionlab, Hiroshima, Japan) and stored at −80 °C. We prepared cryo-sections of 7 µm thickness using Kawamoto cryofilm type 2C (Sectionlab, Hiroshima, Japan). Prior to each staining procedure, slices were dried for 20 min at RT and at the final step covered with DPX Mountant (Sigma-Aldrich Chemie GmbH, Munich, Germany).

Hematoxylin and Eosin (H&E) staining was conducted using the following protocol: fixing with 4% PFA for 10 min, washing with ddH_2_O for 5 min, first staining with Harris’s hematoxylin for 7 min (Merck, Darmstadt, Germany), washing twice with ddH_2_O, differentiating in 0.25 mL HCl solution (37% HCl, Merck, Darmstadt, Germany) in 100 mL of 70% ethanol. After washing with tap water for 10 min, second staining with Eosin (0.2%, 2 min; Chroma Waldeck GmbH & Co KG, Münster, Germany) was performed, differentiation step in 96% ethanol, followed by 100% ethanol (2 × 2 min) and xylol (2 × 2 min) treatments.

Von Kossa Toluidine blue staining was performed according to the following protocol: fixing with 4% PFA (10 min), washing with ddH_2_O (5 min), staining in silver nitrate solution (3% in ddH_2_O, 10 min), washing with ddH_2_O, staining in sodium carbonate/formaldehyde solution (2 min), washing with ddH_2_O, followed by a staining step in sodium thiosulfate solution (5% in ddH_2_O, 5 min). After washing with tap water for 10 min, slices were washed with ddH_2_O, counter stained in Toluidine blue solution for 8 min, washed with ddH_2_O, differentiated in 70% ethanol, 100% ethanol and fixed in xylol (each 2 × 2 min).

Alcian Blue staining was performed according to the following protocol: fixing with 4% PFA (10 min), washing with ddH_2_O (5 min), treating with 3% acetic acid (3 min), staining step in 1% Alcian Blue for 30 min (Sigma-Aldrich Chemie GmbH, Munich, Germany), washing in 3% acetic acid (pH 2.5), washing with ddH_2_O, staining step in nuclear fast red aluminum sulfate for 4 min, washing with ddH_2_O, followed by 80%, 96% and 100% ethanol (2 min each) and fixing with xylol (2 × 2 min).

### 4.9. Immunohistochemistry

Immunohistochemistry was performed according to the following protocol: rehydrating with PBS (10 min), blocking with 3% H_2_O_2_ (30 min), washing with PBS (5 min), blocking with 5% normal horse serum (Vector Laboratories, Burlingame, CA, USA) in 2% bovine serum albumin (BSA, Sigma-Aldrich Chemie GmbH, Munich, Germany)/PBS, first incubation step with primary antibody for collagen type I (1:500, Abcam plc, Cambridge, UK) or collagen type II (1:10, Quartett GmbH, Berlin, Germany) at 4 °C overnight, washing in PBS (2 × 5 min), second incubation step with 2% biotinylated horse anti-mouse IgG antibody (secondary antibody, Vector Laboratories, Burlingame, CA, USA) diluted in 5% normal horse serum/2% BSA/PBS for 30 min and washing in PBS (2 × 5 min). After incubation with Vecastain^®^ Elite^®^ ABC HRP Kit (Vector Laboratories, Burlingame, CA, USA) for 50 min, slices were washed with PBS (2 × 5 min), incubated with DAB Peroxidase HRP Substrate Kit (microscopic control, Vector Laboratories, Burlingame, CA, USA), thus the reaction was stopped with PBS, followed by washing with ddH_2_O, counter staining step in Mayer’s hematoxylin (2 min, Sigma-Aldrich Chemie GmbH, Munich, Germany), washing in tap water (10 min) and finally washing in ddH_2_O.

### 4.10. Immunofluorescence Staining

Immunofluorescence staining was performed in a dark, humid chamber at RT. First, slices were air dried and then rehydrated with PBS for 10 min. Subsequently, unspecific binding sites were blocked with PBS/5% FCS for 30 min. Afterwards, primary antibody was diluted in PBS/5% FCS/0.1% Tween^®^ 20 (Qbiogene Inc., Carlsbad, CA, USA) and incubated according to the manufacturer’s instructions, followed by washing with PBS/0.1% Tween^®^ 20 (3×). Secondary antibody was diluted in PBS/5% FCS/0.1% Tween^®^ 20 and applied for 2 h, washed with PBS/0.1% Tween^®^ 20 (3×) and nuclei staining was performed using 4′,6-diamidino-2-phenylindole (DAPI; 1 µg/mL diluted in PBS/5% FCS/0.1% Tween^®^ 20, 15 min). After bubble-free covering with FluoroMount (Sigma‑Aldrich Chemie GmbH, Munich, Germany) covering medium, imaging was performed with the fluorescence microscope BZ-9000 (Keyence). Image analysis was performed using ImageJ. Primary and secondary antibodies used for immunofluorescence staining are listed in Table 2.

TUNEL assay (Sigma‑Aldrich Chemie GmbH, Munich, Germany) was performed to detect apoptotic cells. As a positive control, slices were treated with desoxyribonuclease (DNase) I (0.34 Kunitz units, Qiagen GmbH, Hilden, Germany) for 10 min and washed twice with PBS. Staining was performed with TUNEL reaction mixture (5 µL TUNEL enzyme + 45 µL TUNEL label) for 1 h at 37 °C and then washed twice with PBS. A negative control was incubated with TUNEL label without TUNEL enzyme.

### 4.11. Image Analysis with ImageJ

Evaluation of immunofluorescence images was performed using FIJI ImageJ 1.52p (National Institutes of Health, Bethesda, MD, USA). First, a free hand selection tool was used to define the region of interest (ROI) representing the total area (t.a.). The outer area (o.a.) was determined by a reduction of the diameter by 0.95 for X- and Y-axes. Another reduction of the diameter by 0.5 for X- and Y-axes leads to the middle area (m.a.; outer area subtracted from total area, diameter reduced by 0.5 for X- and Y-axes) and inner area (i.a.; remaining inner part). The positive stained areas were defined using the color threshold tool. Finally, the cell count was performed with a combination of the Find Maxima tool in ImageJ and manual counting.

### 4.12. In Vitro µCT

TBBCs were scanned at a nominal resolution of 4–5 μm, with a Bruker SkyScan 1172 high-resolution microCT (Bruker, Kontich, Belgium). X-ray tube voltage was of 80 kV and a 0.5 mm aluminum filter was employed. The scan orbit was 360 degrees with a rotation step of 0.3 degree. For reconstruction the SkyScan NRecon software was used and Gaussian smoothing, ring artifact reduction, misalignment compensation, and beam hardening correction were applied. CTAn (Bruker MicroCT, Kontich, Belgium) software was used to analyze the total VOI.

### 4.13. RNA Isolation, cDNA Synthesis and qPCR

Total RNA from the 3D components was isolated according to the manufacturer’s instructions using the RNeasy^®^ Fibrous Tissue Mini Kit (Qiagen GmbH, Hilden, Germany) after homogenization with the TissueRuptor II (Qiagen GmbH, Hilden, Germany). Total RNA from monolayer (ML) cultivations was isolated according to the manufacturer’s instructions using the RNeasy^®^ Mini Kit (Qiagen GmbH, Hilden, Germany). RNA concentrations were determined via NanoDrop^®^-ND-1000 Spectrophotometer (Thermo Fisher Scientific, Waltham, MA, USA) and stored at −80 °C. TaqMan^®^ Reverse Transcription Reagents Kit (Applied Biosystems Inc., Foster City, CA, USA) was used for cDNA synthesis with more than 50 ng per reaction whereas Sensiscript Reverse Transcriptase Kit (Qiagen GmbH, Hilden, Germany) was used for cDNA synthesis with less than 50 ng per reaction. Primer were designed using Primer Blast (NCBI, Bethesda, MD, USA). Sequence analyses of qPCR products were confirmed by LGC Genomics GmbH (Berlin, Germany) and evaluated using the Chromas software 2.6.4 (Technelysium Pty Ltd., South Brisbane, Australia). Quantitative PCR (qPCR) was carried out using the DyNAmo ColorFlash SYBR Green qPCR Kit (Thermo Fisher Scientific, Waltham, MA, USA) in the Stratagene Mx3000P^TM^ (Agilent Technologies Inc., Santa Clara, CA, USA). The qPCR was conducted in duplicates with a non-template control (NTC) for each mastermix using the following temperature profile: 7 min initial denaturation at 95 °C, 45 to 60 cycles of 10 s denaturation at 95 °C, 7 s annealing at 60 °C and 9 s elongation at 72 °C. After every run, a melting curve analysis was performed to confirm primer specificity. In cases where the amplification curve did not reach the threshold within the cycles, the value of the maximum cycle number was used. All primers were purchased from TIB Molbiol Berlin, Germany (Table 3).

### 4.14. Statistical Analysis

Statistical analysis was performed using the GraphPad^®^ Prism V.8.4.1 software (GraphPad Software, La Jolla/San Diego, CA, USA). All values are shown as the mean ± SEM if not indicated otherwise. Mann-Whitney U test was applied for independent datasets while dependent datasets were compared by means using the Wilcoxon-signed rank test. Values of *p* < 0.05 were considered as statistically significant.

## 5. Conclusions

We herein describe a novel alternative approach simulating key features of RA including cartilage destruction and subchondral bone erosion in late stages of disease to be used as preclinical drug screening tool. The 3D osteochondral tissue model replicates the interaction of cells within a physiological matrix and environment, the crosstalk between the major resident cells of human cartilage and anabolic bone, and the option to further expand cellular interactions by the application of e.g., leukocytes.

## Figures and Tables

**Table 1:** Human MSCs donor information and conducted experiments.

Donor	Age	Sex	Type of Experiments	Used Methods
MSC 1	62	m	Characterization of TBBCs	LDH, WST-1, gene expression analysis, histology, immunofluorescence
MSC 2	78	m		
MSC 3	56	w		
MSC 4	69	w		
MSC 5	57	m		
MSC 6	74	w		
MSC 7	75	w		LDH, µCT, SEM, Alizarin Red, histology, immunofluorescence
MSC 8	76	w		
MSC 9	77	m		
MSC 10	77	w		
MSC 11	66	m		
MSC 12	53	m		
MSC 13	63	m		
MSC 14	84	w	Characterization of SFCCs	Gene expression analysis, histology
MSC 15	71	w		
MSC 16	66	m		
MSC 17	59	w		
MSC 18	79	m		
MSC 19	78	m		
MSC 20	64	m	Co-cultivation (OTM), proof of OTM experiments	Gene expression analysis, histology
MSC 21	67	w		
MSC 22	72	w		
MSC 23	76	w		
MSC 24	57	m		

**Table 2:** Primary and secondary antibodies used for immunofluorescence staining.

**Primary** **Antibody**	**Dye**	**Host**	**ID**	**Concentration [mg/mL]**	**Company**
Phalloidin	TRITC	-	P1951	1.5	Sigma‑Aldrich
Laminin	-	rabbit	NB300	0.8	Novus Biologicals LLC
Osteopontin	A488	rabbit	ab8448	1	Abcam
Collagen 1	-	mouse	ab6308	1.5	Abcam
Collagen 2	-	mouse	CO072	1	Quartett
**Secondary** **Antibody**	**Dye**	**Host**	**ID**	**Concentration [mg/mL]**	**Company**
Anti-mouse	Biotin	horse	BA-2000	1.5	Vector Laboratories Inc.
Anti-rabbit	A488	goat	A32731	2	Thermo Fisher Scientific

**Table 3:** Sequences of primers, fragment size and GenBank ID used for qPCR.

Gene	Sequence of Forward Primer	Sequence of Reverse Primer	GenBank ID
*EF1A*	GTTGATATGGTTCCTGGCAAGC	TTGCCAGCTCCAGCAGCCT	NM_001402.5
*RUNX2*	TTACTTACACCCCGCCAGTC	TATGGAGTGCTGCTGGTCTG	NM_001015051.3
*SPP1*	GCCGAGGTGATAGTGTGGTT	TGAGGTGATGTCCTCGTCTG	NM_001251830.1
*COL1A1*	CAGCCGCTTCACCTACAGC	TTTTGTATTCAATCACTGTCTTGCC	NM_000088.3
*ON*	ACCAGCACCCCATTGACG	AGGTCACAGGTCTCGAAAAAGC	NM_001309443.1
*SOX9*	CGCCTTGAAGATGGCGTTG	GCTCTGGAGACTTCTGAACGA	NM_000346.3
*PPARG2*	CAAACCCCTATTCCATGCTGTT	AATGGCATCTCTGTGTCAACC	NM_015869.4
*COL2A1*	GTGGGGCAAGACTGTTATCG	AGGTCAGGTCAGCCATTCAG	NM_033150.3
*COL10A1*	CCAGCACGCAGAATCCATCT	TATGCCTGTGGGCATTTGGT	NM_000493.4
*ACAN*	AACGCAGACTACAGAAGCGG	GGCGGACAAATTAGATGCGG	NM_001369268.1
*MMP1*	CTCTGGAGTAATGTCACACCTCT	TGTTGGTCCACCTTTCATCTTC	NM_001145938.2
*MMP3*	ATCCTACTGTTGCTGTGCGT	CATCACCTCCAGAGTGTCGG	NM_002422.5
*MMP13*	TCCTGATGTGGGTGAATACAATG	GCCATCGTGAAGTCTGGTAAAAT	NM_002427.4
*TNF*	GTCTCCTACCAGACCAAG	CAAAGTAGACCCTGCCCAGACTC	NM_000594.4
*IL6*	TACCCCCAGGAGAAGATTCC	TTTTCTGCCAGTGCCTCTTT	NM_001371096.1
*IL8*	GAATGGGTTTGCTAGAATGTGATA	CAGACTAGGGTTGCCAGATTTAAC	NM_000584.4
*LDHA*	ACCCAGTTTCCACCATGATT	CCCAAAATGCAAGGAACACT	NM_005566.4
*VEGFA*	AGCCTTGCCTTGCTGCTCTA	GTGCTGGCCTTGGTGAGG	NM_001025366.3

## Data Availability

The data presented in this study are available on request from the corresponding author. The data are not publicly available due to privacy and ethical restrictions.
